# Cross-Sectional Associations between Mothers and Children’s Breakfast Routine—The Feel4Diabetes-Study

**DOI:** 10.3390/nu13030720

**Published:** 2021-02-24

**Authors:** Natalia Giménez-Legarre, Alba M. Santaliestra-Pasías, Greet Cardon, Rurik Imre, Violeta Iotova, Jemina Kivelä, Stavros Liatis, Konstantinos Makrilakis, Christina Mavrogianni, Tatjana Milenkovic, Anna Nánási, Tsvetalina Tankova, Patrick Timpel, Ruben Willems, Yannis Manios, Luis A. Moreno, on behalf of the Feel4Diabetes-Study Group

**Affiliations:** 1 GENUD (Growth, Exercise, Nutrition and Development) Research Group, Facultad de Ciencias de la Salud, Universidad de Zaragoza, 50009 Zaragoza, Spain; nglegarre@unizar.es (N.G.-L.); lmoreno@unizar.es (L.A.M.); 2 Instituto Agroalimentario de Aragón (IA2), 50013 Zaragoza, Spain; 3 Instituto de Investigación Sanitaria Aragón (IIS Aragón), 50009 Zaragoza, Spain; 4 Centro de Investigación Biomédica en Red de Fisiopatología de la Obesidad y Nutrición (CIBERObn), Instituto de Salud Carlos III, 28029 Madrid, Spain; 5 Department of Movement and Sports Sciences, Ghent University, 9000 Ghent, Belgium; greet.cardon@ugent.be; 6 Department of Family and Occupational Medicine, University of Debrecen, 4032 Debrecen, Hungary; rurik.imre@sph.unideb.hu (R.I.); nanasi.anna@med.unideb.hu (A.N.); 7 Department of Pediatrics, Medical University of Varna, 9002 Varna, Bulgaria; iotova_v@yahoo.com; 8 Public Health Promotion Unit, Finnish Institute for Health and Welfare, 00271 Helsinki, Finland; jemina.kivela@thl.fi; 9 National and Kapodistrian University of Athens Medical School, 11527 Athens, Greece; sliatis@med.uoa.gr (S.L.); kmakrila@yahoo.com (K.M.); 10 Department of Nutrition and Dietetics, School of Health Science and Education, Harokopio University, 17671 Athens, Greece; cmavrog@hua.gr (C.M.); manios.feel4diabetes@hua.gr (Y.M.); 11 Medical Faculty St. Cyril and Methodius, 1000 Skopje, North Macedonia; milenkovic.tatjana@yahoo.com; 12 University Clinic of Endocrinology, Diabetes and Metabolic Disorders, 1000 Skopje, North Macedonia; 13 Department of Endocrinology, Medical University of Sofia, 1431 Sofia, Bulgaria; tankova@iname.com; 14 Department for Prevention and Care of Diabetes, Faculty of Medicine Carl Gustav Carus, Technische Universität Dresden, 01307 Dresden, Germany; patrick.timpel@tu-dresden.de; 15 Department of Public Health and Primary Care, Ghent University, 9000 Ghent, Belgium; Ruben.Willems@ugent.be; 16 Institute of Agri-Food and Life Sciences, Hellenic Mediterranean University Research Centre, 71410 Heraklion, Greece

**Keywords:** breakfast, children, mother, mother´s influence

## Abstract

Positive influences of family members have been associated with a high probability of children’s daily breakfast consumption. Therefore, the aim of this study was to scrutinize the association of breakfast routines between mothers and their children. The baseline data of the Feel4Diabetes-study was obtained in 9760 children (49.05% boys)–mother pairs in six European countries. A parental self-reported questionnaire gauging the frequency of breakfast consumption and of breakfast´ foods and beverages consumption was used. Agreement in routines of mothers and their children’s breakfast consumption was analyzed in sex-specific crosstabs. The relationship of breakfast routine and food groups’ consumption between mothers and their children was assessed with analysis of covariance. The highest proportion of children who always consumed breakfast were those whose mothers always consumed it. Children consuming breakfast regularly had a higher intake of milk or unsweetened dairy products and all kind of cereal products (low fiber and whole-grain) than occasional breakfast consumers (*p* < 0.05). The strong similarity between mothers and children suggests a transfer of breakfast routine from mothers to their children, as a high proportion of children who usually consume breakfast were from mothers also consuming breakfast. All breakfast foods and beverages consumption frequencies were similar between children and their mothers.

## 1. Introduction

Breakfast is an essential part of a healthy diet [1] because it has been associated with general health and well-being in both adults and children [2]. Recent systematic reviews and meta-analyses observed that breakfast consumption was associated with better daily macro- and micronutrient intake in children and adolescents. [3,4]. It has been reported that children and adolescents who ate breakfast regularly had a healthier food pattern compared with breakfast skippers [5]. Moreover, breakfast consumption has been associated with the daily consumption of healthier foods and beverages, such as fruits and vegetables, dairy products, and cereals [4]. Most children who often skip breakfast usually do not meet the daily recommendations for fruits and vegetables [6]. Different studies observed that skipping breakfast also had reduced intakes of many nutrients, like vitamins, minerals, or dietary fiber [7,8], and a high prevalence of nutrient inadequacy [9]. In agreement with this, previous systematic review and meta-analysis observed that skipping breakfast was associated with worse macronutrient intake and lower micronutrient intake [3,4].

Breakfast characteristics have changed over time depending on culture and food availability in different countries [1]. The breakfast routine should be part of a healthy lifestyle, and it is essential to be personalized according to eating preferences, habits, and culture [2]. Omission of breakfast or its irregular consumption has been associated with increased cardiovascular [10,11] or type 2-diabetes risk factors in adults and children [12,13]. Skipping breakfast has also been associated with increased appetite and a high risk of weight gain [14,15] and, as a consequence, overweight and obesity [16]. Breakfast consumption plays a protective role in preventing excess adiposity in children and adolescents [17,18], and in adolescents, it has been associated with high quality of daily nutrition also [4,8]. On the other hand, in children, it was observed that those who ate breakfast daily, particularly a high fiber cereal breakfast, had lower levels of insulin resistance [8]. Furthermore, in European adolescents, it was observed that regular breakfast consumption was associated with a healthier cardiovascular profile [19].

The social environment supporting children and adolescents has an impact on lifestyle behaviors, such as diet [20,21]. Positive influences from both friends and family members are found to be associated with a high probability of daily breakfast consumption [21]. In Australian families, those parents who shared meals with their children promoted healthy behaviors in their families [20]. Parental breakfast consumption has been associated with adolescent´s breakfast consumption routine in a recent review [22]. In children and adolescents, in both sexes, some studies observed that maternal encouragement and eating behaviors influenced regular consumption of breakfast [23,24]. In adolescents, it was observed that girls tended to skip breakfast more often than boys [25]. Furthermore, girls who perceived their family relationships as negative were more likely to skip meals [26]. In a longitudinal study, it was found that family cohesion was positively associated with girl´s breakfast consumption over a 10-year period [27]. However, to our knowledge, there is no information on associations between mothers and their children on breakfast routines and food consumption for breakfast.

The aim of our study was therefore to describe the breakfast routine in children and their mothers, analyzing (i) whether there is an association of breakfast habits between mothers and their children by sex, and (ii) whether there is an association of foods and beverages consumption at breakfast in between them. The study comprised a large sample of families from six European countries.

## 2. Materials and Methods

### 2.1. Study Design and Data Collection

The current study considered the baseline data of the Feel4Diabetes-study, which was an intervention study aiming to develop, implement, and evaluate an evidence-based and potentially cost-effective and scalable intervention program to prevent type 2 diabetes across Europe, primarily focusing on families from vulnerable groups. The Feel4Diabetes-study, which had a cluster-randomized design, was conducted in six European countries, representing high income (Belgium and Finland), low/middle income countries (Bulgaria and Hungary), and high-income countries under austerity measures (Greece and Spain) [28]. Recruitment was based on a standardized, multi-stage sampling procedure and was conducted in six European countries. Participating countries were categorized into three socioeconomic levels according to the World Bank country classification. In each country, children, as well as their parents, attending the first three grades of primary schools located in the selected vulnerable areas were recruited to the study [28,29]. The baseline survey was conducted in the scholar year 2015–2016. Baseline measurements were conducted between April and June 2016. However, for three countries (Finland, Hungary, and Bulgaria) baseline measurements were extended during August and September 2016) to achieve the sample size. To account for seasonal variations, and to minimize the impact on each individual over time, follow-up measurements were conducted as close to the date of the baseline measurements as possible [28].

From the total Feel4Diabetes-study sample (*n* = 11,396 families), only mothers and their children with available information on breakfast consumption were considered (*n* = 9760) in this analysis. 

The Feel4Diabetes-study adhered to the Declaration of Helsinki and the conventions of the Council of Europe on human rights and biomedicine and was approved by each local ethical committee. More specifically, in Belgium, by the Medical Ethics Committee of the Ghent University Hospital (ethical approval code: B670201524237; date approval: 21/04/15); in Bulgaria, by the Ethics Committee of the Medical University of Varna (ethical approval code: 52/10-3-201r; date approval: 10/03/16) and the Municipalities of Sofia and Varna, as well as the Ministry of Education and Science local representatives; in Finland, by the hospital district of Southwest Finland ethical committee (ethical approval code: 174/1801/2015; date approval: 13/03/15); in Greece, by the Bioethics Committee of Harokopio University (ethical approval code: 46/3-4-2015; date approval: 03/04/15) and the Greek Ministry of Education; in Hungary, by the National Committee for Scientific Research in Medicine (ethical approval code: 20095/2016/EKU; date approval: 29/03/16); in Spain, by the Clinical Research Ethics Committee and the Department of Consumers´ Health of the Government of Aragón (ethical approval code: CP03/2016; date approval: 08/04/15). Participants received an information letter in which they were informed about the purpose of the study. Written and signed informed consent was obtained from the parents/caregivers [28].

### 2.2. Maternal Education

Maternal education was selected as a marker of the socioeconomic status of the participating families. It was obtained by a self-administered questionnaire completed by mothers. This was asked on a six-point scale ranging from “less than 7 years” to “more than 16 years” of education.

### 2.3. Anthropometric Measures

Mother´s body mass index (BMI) was calculated based on their self-reported weight and height. Children were measured at schools by trained researchers [30]. Bodyweight was measured in light clothing and without shoes using an electronic scale (Type SECA 813; SECA, Hamburg, Deutschland), and body height was measured with a telescopic height-measuring stadiometer (Type SECA 217; SECA, Hamburg, Deutschland). Two readings were obtained out of each measurement and the mean was used for the analysis. A third measurement was also taken if the previous two measurements differed >100 g for weight or >1 cm for height. BMI was calculated from weight and height (kg/m^2^), and also specific sex- and age- BMI z-score (zBMI) were calculated according to Cole et al. [31].

### 2.4. Breakfast Consumption and Food and Beverages Breakfast Consumption

The family´s energy balance-related behaviors questionnaire was developed in order to assess habits related to diet and physical activity (total time devoted to physical activity and screen activities). This questionnaire has previously been validated for its use in European countries [32]. The questionnaire was developed to be filled in by one of the parents or caregivers, who completed it both for him/herself and their child. Parents or caregivers were contacted through their children´s school. An information letter regarding the objectives of the study and instructions about how to complete the questionnaires was sent. Questionnaires were delivered to the parents via the school and completed by them at home. Researchers were available to answer any doubt by e-mail or phone.

Parents also responded which one of them fulfilled the questionnaire. Only questionnaires filled in by mothers were used in this study as they represent the great majority (88.5%).

#### 2.4.1. Breakfast Consumption

The frequency of mothers´ and children´s breakfast consumption was evaluated by the question “On how many days do you/does your child usually eat breakfast (a) on weekdays, and (b) on weekend days?”. For weekdays, there were six possible answers ranging from “never/rarely” to “five days/week”. For weekend days, there were three possible answers ranging from “never/rarely” to “two days/week”. These variables were grouped and categorized into three groups: (a) Breakfast skippers (0–1 day/week), (b) Occasional breakfast consumers (2–5 days/week), and (c) breakfast consumers (6–7 days/week).

#### 2.4.2. Foods and Beverages Breakfast Consumption

The frequency of mothers´ and children´s foods and beverages breakfast consumption was evaluated by the question, “Having in mind the previous week, how often did you and your child consume the following foods/beverage groups as part of your and his/her breakfast. Breakfast products chosen for the questionnaire were related to the goals of Feel4Diabetes intervention. Eight food and beverage groups were included: fruits, berries and vegetables, fruit juices fresh no sugar, soft drinks and sugar juices, milk or dairy products unsweetened, milk or dairy products sweetened, sweet or salty pastries, low fiber cereal products, and whole-grain cereal products. Each group had four possible answers ranging from “0 days/week” to “7 days/week”. These variables were summed up and categorized into three groups for each food and beverage group: (a) never (0 days), (b) sometimes (1–4 days/week), and (c) always (5–7 days/week).

### 2.5. Statistical Analysis

SPSS Statistics for Windows, Version 21.0, released in 2012 (IBM Corp., Armonk, NY, USA) was used to analyze the data. According to the nature of the studied variables, chi-square test for categorical variables and analysis of variance (ANOVA) for continuous variables were used to compare gender specific sample characteristics. Crosstabs, including chi-square tests for mothers’ and children’s breakfast consumption, were used to analyze the breakfast routine and the frequency of food and beverage group’s breakfast consumption. Analysis of covariance (ANCOVA) was used to analyze the food and beverage groups’ consumption of mothers and children in those who occasionally or usually consume breakfast. ANCOVA analysis between food and beverage groups’ consumption of mother were adjusted by country, maternal education, and maternal BMI; ANCOVA analysis between food and beverage groups’ consumption of children were adjusted by country, maternal education, and child’s zBMI. Microsoft Excel version 14.7.0 was used to develop all graphics and figures. All statistical tests were stratified by child´s sex, and *p*-values lower than 0.05 were considered statistically significant. 

## 3. Results

In total, 9760 children (49.05% boys) and their mothers were included in the analysis. Table 1 presents sex-specific sociodemographic characteristics and breakfast routine. In the study sample, boys were older than girls.

**Figure 1:**
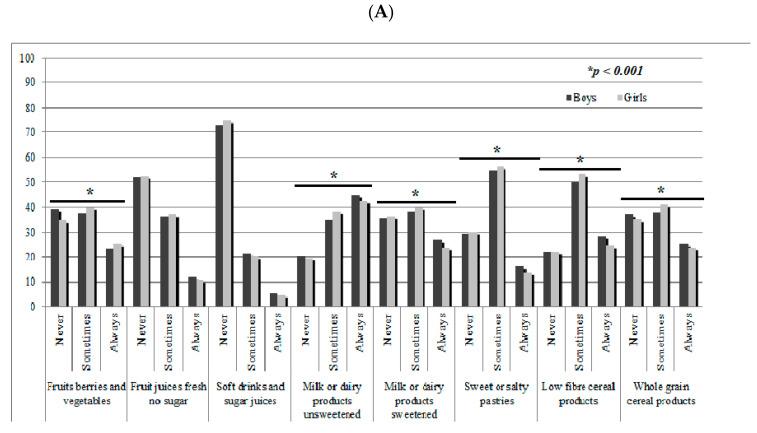

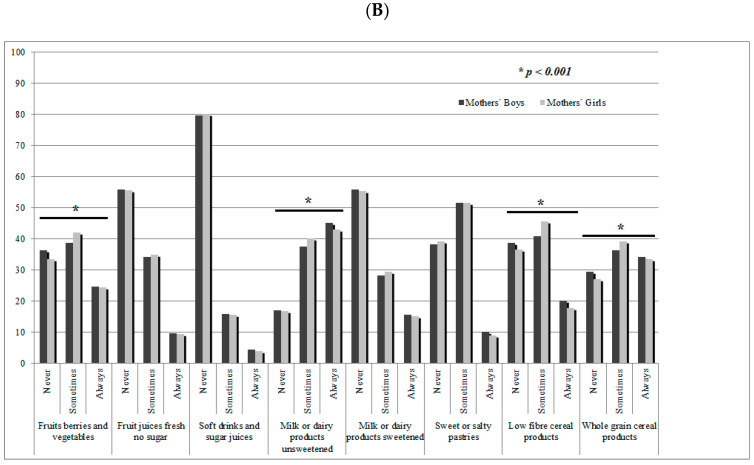
Description of food and beverages consumed at breakfast in children (**A**) and their mothers (**B**) who occasionally or usually consumed breakfast by gender. * *p* values from chi-square indicate statistical significance between genders. Frequency of food and beverage groups’ consumption: Never (0 times/week), Sometimes (1–4 times/ week), Always (5–7 times/week).

Figure 1 presents the description of food and beverages consumed at breakfast in children (1A) and their mothers (1B) who occasionally or usually consumed breakfast by gender. In children (Figure 1A), there were significant differences between sexes in the consumption of fruits, berries, and vegetables, milk or dairy products unsweetened and sweetened, sweet or salty pastries, and low fiber and whole-grain cereal products. A high proportion of children always consumed milk or dairy products at breakfast (unsweetened: 45.0% of the boys and 42.2% of the girls; sweetened: 26.8% of the boys and 23.8% of the girls). Around one-quarter of the sample consumed cereals at breakfast (low fiber cereals, 28.2% of boys and 24.5% of girls; whole-grain cereal, 25.2% of boys and 23.6% of girls). Regarding the mothers’ consumption of food and beverages at breakfast (Figure 1B) in those who occasionally or usually consumed breakfast, significant differences between genders were found in the consumption of fruits, berries, and vegetables, milk or dairy products unsweetened and low fiber and whole-grain cereal products. A high proportion of mothers always consumed milk or dairy products unsweetened at breakfast (45.2% of boys´ mothers, 43.0% of girls´ mothers). On the other hand, 24.8% of the boys´ mothers and 24.4% of the girls´ mothers usually consume fruit, berries, and vegetables at breakfast. For the consumption of cereal products, high variability was found. Low fiber cereals were consumed by 20.2% of the boys´ mothers and 17.8% of the girls´ mothers, however, whole-grain cereals were always consumed by 34.2% and 33.5% of the boys´ and girls´ mothers, respectively. 

Table 2 and Table 3 present the description of food and beverages consumed at breakfast in children (Table 2) and their mothers (Table 3) who occasionally or usually consumed breakfast by gender. In both, children and their mothers, there were significant differences between occasional breakfast consumers and breakfast consumers in all food and beverages. In children, in both genders, the most consumed foods groups and beverages at breakfast by breakfast consumers were milk or dairy products unsweetened (45.8% of the boys and 42.7% of the girls) and low fiber cereal products (28.8% of the boys and 25.2% of the girls). In boys, 26.4% of the breakfast consumers consumed milk or dairy products sweetened at breakfast. In girls, 25.9% of the breakfast consumers consumed fruits, berries, and vegetables at breakfast. In mothers, the most consumed foods groups and beverages at breakfast by breakfast consumers were milk or dairy products unsweetened (48.8% of the boys’ mothers breakfast consumers and 46.4% of the girls’ mothers breakfast consumers), low fiber cereal products (41.1% of the boys’ mothers breakfast consumers and 35.0% of the girls’ mothers breakfast consumers) and whole-grain cereals products (39.3% of the boys’ mothers breakfast consumers and 38.6% of the girls’ mothers breakfast consumers).

Table 4 presents the proportion of mothers and their children´s breakfast routine. The highest proportion was for children who usually consumed breakfast in concordance with their mothers (67.95% in boys; 65.65% in girls). At the opposite, only 0.44% of boys and 0.60% of girls of the total sample were in concordance with their mothers as breakfast skippers. Also, 3.45% of boys and 3.48% of girls whose mothers occasionally consumed breakfast were occasional breakfast consumers as well. Other possible combinations were observed, 0.06% of the boys and 0.14% of the girls whose mothers always consumed breakfast were breakfast skippers, and 1.80% of the boys and 2.39% of the girls whose mothers always consumed breakfast were occasional breakfast consumers. 

Mean scores of food and beverage consumption (times/week) at breakfast in children who occasionally or usually consumed breakfast are shown in Table 5. Firstly, the analyses were performed without stratification by sex (table not shown), however, it was observed some differences between sexes and results were presented stratified by sex. In both boys and girls, breakfast consumers had a higher intake of milk or dairy products unsweetened, low fiber cereal and whole-grain cereals products at breakfast than occasionally breakfast consumers (*p* < 0.001). Girls usually consuming breakfast had a higher intake of fruits, berries and vegetables, and a lower intake of soft drinks and sugar juices (*p* < 0.001) at breakfast compared to girls occasionally consuming breakfast. 

Table 6 presents the mean scores of food and beverage group´s consumption (times/week) at breakfast in mothers who occasionally or usually consumed breakfast. Mothers (either boys and girls) usually consuming breakfast had a higher intake of fruits, berries and vegetables, milk, and dairy unsweetened and sweetened products, low fiber and whole-grain cereal products (*p* < 0.001), and fresh and unsweetened fruit juices (*p* < 0.001) at breakfast compared to mothers occasionally consuming breakfast. These mothers who usually consumed breakfast also had a lower intake of soft drinks and sugar juices compared to mothers who occasionally consumed breakfast (*p* < 0.001).

Table 7 presents the cross-tabulation between the frequency of food and beverage consumption at breakfast between mothers and their children. In both, boys and girls, breakfast food and beverages consumption were in concordance with their mothers’ consumption in all food and beverage groups. In both sexes, close to 60% and 50% of children who always consumed fruits, berries, and vegetables, and fresh and unsweetened fruit juices were those whose mothers always consumed the same food and beverages groups (*p* < 0.001). Regarding consumption of soft drinks, more than 90% of children who never consume them were those whose mothers never consume them (*p* < 0.001). The high proportion of children who always consumed milk or dairy products unsweetened or sweetened, and low fiber or whole-grain cereals products were those whose mothers always consume them (*p* < 0.001). The high proportion of children who never consumed sweets or salty pastries were those whose mothers never consume it (*p* < 0.001).

## 4. Discussion

The most important finding of the current study is that children´s breakfast routine is associated with mothers´ breakfast routine, independently of the children’s gender. To the best of our knowledge, this is the first study to examine the associations between the breakfast routine of children and their mothers, analyzing breakfast habits, and also the foods and beverages consumed at breakfast. 

Our findings showed that 92.4% of the boys and 91.8% of the girls were breakfast consumers. During childhood, the proportion of breakfast consumption in six countries was similar to our study [33]. Regarding mothers, our results showed that 69.8% of boys´ mothers and 68.2% of girls´ mothers were breakfast consumers, which is a lower prevalence than the one observed in the International Breakfast Research Initiative, in which it was observed that between 76–95% of young adults from different countries usually consume breakfast [33]. 

In our study, we observed that in both sex groups, breakfast routine in terms of frequency of breakfast consumption and foods and beverages consumed at breakfast were in agreement with their mother´s breakfast routine. The highest proportion of breakfast skippers were observed in those whose mothers usually skip breakfast, while the highest proportion of breakfast consumers were those whose mothers usually consume breakfast. In agreement with our research, a previous study showed that children´s dietary habits, which include breakfast consumption, are associated with their parents’ dietary habits [34]. Recent research observed a significant relation between the breakfast consumption frequency of the father or the mother and breakfast frequency of children [35]. Furthermore, strong parental support for breakfast as the main daily meal was significantly associated with children´s daily breakfast intake [36]. Another study developed in European children of similar ages that our study suggested that children were more likely to consume breakfast daily because they had high parental modeling [21]. Parents influence children´s diets, especially during shared family mealtimes [20]. Furthermore, some authors concluded that food behaviors are developed and reinforced by the family food environment [20,37].

Regarding the foods and beverages consumed at breakfast, in both, boys and girls, breakfast consumers had a higher intake of milk or unsweetened dairy products and cereal products (low fiber and whole-grain) compared to occasionally breakfast consumers. In agreement with our results, different studies showed that ready-to-eat cereals, milk, and bread are the main components in children and adolescents´ breakfast in Europe [38,39,40]. Indeed, a previous systematic review observed that breakfast based on ready-to-eat cereals is the most commonly consumed type of breakfast by children and adolescents, and furthermore, those who usually consume breakfast showed a higher daily intake of milk and dairy products and cereals than breakfast skippers [4]. Regarding mothers, the results showed a higher intake of fruits, berries and vegetables, fresh and unsweetened fruit juices, milk and dairy products (sweetened and unsweetened), and cereal products (low fiber and whole-grain). This conforms to the findings of a previous study that showed a higher variety of foods and beverages consumed at breakfast by adults [41]. 

Our findings showed that in both genders, breakfast foods and beverages consumption agreed with their mothers´ consumption, being the high proportion of children who always consumed fruits, berries, and vegetables those whose mothers always consumed this type of products. A previous longitudinal study showed that mothers´ and children´s food preferences were significantly associated, and for this reason, those foods and beverages which are disliked by mothers tended not to be offered to children [42]. It was observed that family mealtime routine and parental monitoring during childhood may provide a supportive food environment that promotes children´s overall diet quality [43]. This could be possible because it was observed that parents´ dietary and physical activity practices shape their children´s nutritional and activity patterns [44,45]. Children develop preferences that affect their food selections due to the establishment of eating practices that contribute to lifelong nutritional habits and overall health during childhood. For this reason, parents are responsible for modeling healthy food choices and dietary practices, which shape children’s food preferences and eating behaviors [46,47]. 

Our study is characterized by a number of strengths and limitations. Firstly, it is necessary to take into consideration the cross-sectional design, from which no causal conclusions can be drawn. Second, the term “breakfast consumption” includes a variety of definitions, and nowadays, there is no consensus regarding the definition of breakfast consumption. Third, the questionnaires were self-reported, and it may have been possible that mothers overestimated the child´s and their own consumption. Fourth, we only analyzed mothers´ information because most of the participating parents are women (88.5% women; 10.6% men), and previous authors concluded that especially the mother’s role is of importance to improve children´s diet quality [48,49,50]. Fifth, the frequency of consumption of selected groups of products may differ depending on the season. For three countries (Finland, Hungary, and Bulgaria) baseline measurements were extended during different months to achieve the sample size. To account for seasonal variations and to minimize the impact on each individual overtime, follow-up measurements were conducted as close to the date of the baseline measurements as possible. Furthermore, the study design is a cluster randomized clinical trial, including an overall European sample, but the specifics country cohorts were not representative of the general population. Also, the aim of the study was not to compare the results between countries; however, the major strength of our study is the large sample of children and its geographical distribution across Europe. Moreover, questionnaires have been designed under standardized procedures for a multi-country population, and these have been tested for their reliability in European countries [32].

## 5. Conclusions

In the present study, we analyzed the breakfast routine in a large sample of European children and their mothers. The highest proportion of children was breakfast consumers. Furthermore, a high percentage of the mothers also were breakfast consumers. The highest proportion of children who usually consumed breakfast was found in those whose mothers were breakfast consumers. Also, those food and beverage groups consumed by mothers at breakfast were the same that their children consumed at this eating occasion. Our results may suggest a clear transfer of breakfast routine from the mother to their children, although our analyses do not allow causal inferences. Mothers might be an important influence regarding children’s breakfast consumption, in terms of behavior and also in terms of quality of breakfast. Furthermore, during childhood, breakfast is an established habit; however, it may be lost during adolescence because, at that age, each individual reaffirms their personality and makes their own decisions about eating habits, choices, and preferences. However, more studies are needed to investigate the impact of parents on children´s breakfast routine (frequency and foods and beverages consumed). 

## Figures and Tables

**Table 1:** Descriptive characteristics of the sample of European children from the Feel4Diabetes study (*n* = 9760).

	Boys (*n* = 4788)	Girls (*n* = 4972)	*p* *
Age (years) mean (CI 95%)	8.22 (8.19; 8.24)	8.18 (8.15; 8.20)	**0.050**
Mothers’ Education *n* (%)			
≤12 years	1268 (26.5)	1369 (27.5)	0.126
>12 years	3520 (73.5)	3603 (72.5)	
Country *n* (%)			
Belgium	699 (14.6)	711 (14.3)	0.073
Finland	641 (13.4)	657 (13.2)	
Greece	890 (18.6)	932 (18.7)	
Hungary	720 (15.0)	794 (16.0)	
Bulgaria	1257 (26.3)	1365 (27.5)	
Spain	581 (12.1)	513 (10.3)	
Mothers’ Breakfast Consumption *n* (%)			
Breakfast skippers	575 (12.0)	662 (13.3)	0.114
Occasionally breakfast consumers	871 (18.2)	920 (18.5)	
Breakfast consumers	3342 (69.8)	3390 (68.2)	
Children’s Breakfast Consumption *n* (%)			
Breakfast skippers	30 (0.6)	39 (0.8)	0.411
Occasionally breakfast consumers	332 (6.9)	369 (7.4)	
Breakfast consumers	4426 (92.4)	4564 (91.8)	

* Significant *p* values are shown in bold font. Abbreviations: 95% CI, 95% confidence intervals. Breakfast consumption categories: Breakfast skippers, children/mothers who usually consume breakfast 0–1 days per week; Occasional breakfast consumers, children/mothers who usually consume breakfast 2–5 days per week; Breakfast consumers, children/mothers who usually consume breakfast 6–7 days per week.

**Table 2:** Description of food and beverages consumed at breakfast in children who occasionally or usually consumed breakfast.

		Boys Occasional Breakfast Consumers	Boys Breakfast Consumers	*p* *	Girls Occasional Breakfast Consumers	Girls Breakfast Consumers	*p* *
Fruits, berries and vegetables	Never	131 (33.3)	1966 (38.7)	**0.002**	112 (25.3)	1783 (34.4)	**<0.001**
	Sometimes	181 (46.1)	1889 (37.2)		239 (54.0)	2059 (39.7)	
	Always	81 (20.6)	1219 (24.0)		92 (20.8)	1345 (25.9)	
Fruit juices fresh unsweetened	Never	162 (42.0)	2597 (51.3)	**<0.001**	198 (45.6)	2657 (51.4)	**0.017**
	Sometimes	179 (46.4)	1845 (36.4)		191 (44.0)	1917 (37.1)	
	Always	45 (11.7)	621 (12.3)		45 (10.4)	594 (11.5)	
Soft drinks and sugar juices	Never	219 (57.6)	3630 (72.2)	**<0.001**	234 (54.7)	3806 (74.4)	**<0.001**
	Sometimes	126 (33.2)	1104 (21.9)		150 (35.0)	1038 (20.3)	
	Always	35 (9.2)	297 (5.9)		44 (10.3)	274 (5.4)	
Milk or dairy products unsweetened	Never	77 (19.9)	1017 (20.1)	**<0.001**	71 (16.2)	1005 (19.4)	**<0.001**
	Sometimes	175 (45.2)	1728 (34.1)		231 (52.7)	1962 (37.9)	
	Always	135 (34.9)	2319 (45.8)		136 (31.1)	2209 (42.7)	
Milk or dairy products sweetened	Never	106 (27.6)	1793 (36.2)	**<0.001**	138 (32.0)	1839 (36.5)	**0.001**
	Sometimes	192 (50.0)	1850 (37.4)		209 (48.5)	2000 (39.7)	
	Always	86 (22.4)	1304 (26.4)		84 (19.5)	1202 (23.8)	
Sweet or salty pastries	Never	66 (17.1)	1452 (28.9)	**<0.001**	86 (19.7)	1476 (28.9)	**<0.001**
	Sometimes	255 (66.2)	2753 (54.8)		281 (64.4)	2892 (56.6)	
	Always	64 (16.6)	816 (16.3)		69 (15.8)	742 (14.5)	
Low fiber cereal products	Never	86 (22.5)	1122 (22.3)	**0.022**	87 (19.9)	1134 (22.3)	**0.028**
	Sometimes	210 (55.0)	2455 (48.9)		259 (59.1)	2676 (52.6)	
	Always	86 (22.5)	1448 (28.8)		92 (21.0)	1281 (25.2)	
Whole-grain cereal products	Never	172 (45.7)	1934 (38.7)	**<0.001**	183 (43.3)	1842 (36.4)	**<0.001**
	Sometimes	160 (42.6)	1835 (36.7)		193 (45.6)	2073 (40.9)	
	Always	44 (11.7)	1230 (24.6)		47 (11.1)	1151 (22.7)	

* *p* values from chi-square indicate statistical significance between genders. Frequency of food and beverage groups’ consumption: Never (0 times/week), Sometimes (1–4 times/ week), Always (5–7 times/week).

**Table 3:** Description of food and beverages consumed at breakfast in mothers who occasionally or usually consumed breakfast.

		Boys’ Mothers Occasional Breakfast Consumers	Boys’ Mothers Breakfast Consumers	*p* *	Girls’ Mothers Occasional Breakfast Consumers	Girls’ Mothers Breakfast Consumers	*p* *
Fruits, berries and vegetables	Never	350 (34.8)	1394 (36.8)	**<0.001**	346 (33.1)	1290 (33.8)	**<0.001**
	Sometimes	482 (48.0)	1403 (37.1)		536 (51.3)	1535 (40.2)	
	Always	173 (17.2)	989 (26.1)		162 (15.5)	991 (26.0)	
Fruit juices fresh unsweetened	Never	527 (53.1)	2089 (55.5)	**0.006**	539 (52.5)	2103 (55.4)	**<0.001**
	Sometimes	386 (38.9)	1286 (34.2)		420 (40.9)	1310 (34.5)	
	Always	79 (8.0)	389 (10.3)		68 (6.6)	383 (10.1)	
Soft drinks and sugar juices	Never	650 (67.3)	3007 (80.6)	**<0.001**	701 (69.7)	3007 (80.5)	**<0.001**
	Sometimes	251 (26.0)	560 (15.0)		226 (22.5)	579 (15.5)	
	Always	65 (6.7)	163 (4.4)		79 (7.9)	151 (4.0)	
Milk or dairy products unsweetened	Never	200 (20.1)	618 (16.3)	**<0.001**	197 (18.9)	651 (17.0)	**<0.001**
	Sometimes	515 (51.8)	1327 (34.9)		536 (29.5)	1400 (36.6)	
	Always	279 (28.1)	1852 (48.8)		307 (29.5)	1777 (46.4)	
Milk or dairy products sweetened	Never	524 (52.9)	2083 (55.8)	**<0.001**	541 (52.7)	2071 (54.7)	**<0.001**
	Sometimes	357 (36.1)	1023 (27.4)		377 (36.7)	1080 (25.8)	
	Always	109 (11.0)	630 (16.9)		108 (10.5)	637 (16.8)	
Sweet or salty pastries	Never	273 (27.2)	1555 (40.9)	**<0.001**	310 (29.7)	1584 (41.3)	**<0.001**
	Sometimes	644 (64.1)	1853 (48.7)		638 (61.2)	1892 (49.3)	
	Always	88 (8.8)	394 (10.4)		95 (9.1)	362 (9.4)	
Low fiber cereal products	Never	345 (34.4)	1453 (38.4)	**0.001**	353 (34.0)	1387 (36.6)	**0.005**
	Sometimes	476 (47.5)	1556 (41.1)		520 (50.0)	1691 (35.0)	
	Always	181 (18.1)	775 (20.5)		166 (16.0)	716 (18.9)	
Whole-grain cereal products	Never	420 (41.7)	1010 (26.5)	**<0.001**	410 (39.6)	944 (24.6)	**<0.001**
	Sometimes	469 (46.5)	1302 (34.2)		512 (49.5)	1407 (36.7)	
	Always	119 (11.8)	1495 (39.3)		113 (10.9)	1479 (38.6)	

* *p* values from chi-square indicate statistical significance between genders. Frequency of food and beverage groups’ consumption: Never (0 times/week), Sometimes (1–4 times/ week), Always (5–7 times/week).

**Table 4:** Crosstab between mothers´ and boys´ and girls´ breakfast routine.

	**Boys´ Breakfast Consumers**	**Boys´ Occasional Breakfast Consumers**	**Boys´ Breakfast Skippers**	**Total**	*p* *
Mothers´ breakfast consumers	3253 (67.95)	86 (1.80)	3 (0.06)	3342 (69.81)	**<0.001**
Mothers´ occasional breakfast consumers	700 (14.61)	165 (3.45)	6 (0.12)	871 (18.18)	
Mothers´ breakfast skippers	473 (9.87)	81 (1.70)	21 (0.44)	575 (12.01)	
Total	4426 (92.43)	332 (6.95)	30 (0.62)	4788 (100)	
	**Girls´ Breakfast Consumers**	**Girls´ Occasional Breakfast Consumers**	**Girls´ Breakfast Skippers**	**Total**	*p* *
Mothers´ breakfast consumers	3264 (65.65)	119 (2.39)	7 (0.14)	3390 (68.18)	**<0.001**
Mothers´ occasional breakfast consumers	745 (14.98)	173 (3.48)	2 (0.04)	920 (18.5)	
Mothers´ breakfast skippers	555 (11.18)	77 (1.54)	30 (0.60)	662 (13.32)	
Total	4564 (91.81)	369 (7.41)	39 (0.78)	4972 (100)	

* Significant *p* values are shown in bold font. Breakfast consumers categories: Breakfast skippers, children/mother who usually consume breakfast 0–1 days/week; Occasional breakfast consumers, children/mother who usually consume breakfast 2–5 days/week; Breakfast consumers, children/mother who usually consume breakfast 6–7 days/week.

**Table 5:** Analysis of covariance between food and beverage consumption at breakfast and breakfast routine in children, stratified by gender.

Children’s Foods and Beverage Groups Consumption at Breakfast (Times per week)	Boys’ Breakfast Routine			Girls’ Breakfast Routine		
	Occasionally Breakfast Consumers	Breakfast Consumers		Occasionally Breakfast Consumers	Breakfast Consumers	
	Mean (95% CI)	Mean (95% CI)	*p* *	Mean (95% CI)	Mean (95% CI)	*p* *
Fruits berries and vegetables	2.37 (2.07, 2.66)	2.55 (2.48, 2.62)	0.241	2.38 (2.11, 2.65)	2.70 (2.63, 2.77)	**0.021**
Fruit juices fresh unsweetened	1.80 (1.56, 2.04)	1.77 (1.71, 1.83)	0.826	1.58 (1.36, 1.79)	1.75 (1.70, 1.81)	0.127
Soft drinks and sugar juices	1.26 (1.08, 1.45)	1.15 (1.11, 1.20)	0.245	1.49 (1.33, 1.65)	1.08 (1.04, 1.12)	**<0.001**
Milk or dairy products unsweetened	3.20 (2.88, 3.53)	3.97 (3.89, 4.05)	**<0.001**	3.21 (2.92, 3.50)	3.84 (3.76, 3.92)	**<0.001**
Milk or dairy products sweetened	2.59 (2.28, 2.91)	2.80 (2.72, 2.88)	0.213	2.44 (2.16, 2.71)	2.63 (2.56, 2.70)	0.182
Sweet or salty pastries	2.62 (2.38, 2.85)	2.35 (2.29, 2.40)	**0.031**	2.35 (2.14, 2.55)	2.23 (2.17, 2.28)	0.265
Low fiber cereal products	2.54 (2.25, 2.82)	3.07 (2.99, 3.14)	**<0.001**	2.54 (2.29, 2.79)	2.93 (2.86, 2.99)	**0.004**
Whole-grain cereal products	1.99 (1.71, 2.27)	2.66 (2.60, 2.74)	**<0.001**	2.02 (1.77, 2.28)	2.65 (2.59, 2.73)	**<0.001**

* Statistical significance among different breakfast patterns. Significant *p* values are shown in bold font. Abbreviations: 95% CI, 95% confidence intervals. All analyses were adjusted by the following covariates: BMI z-score, country, and maternal education. Breakfast consumers categories: Occasional breakfast consumers, children who usually consume breakfast 2–5 days per week; Breakfast consumers, children who usually consume breakfast 6–7 days per week.

**Table 6:** Analysis of covariance between food and beverage consumption at breakfast and breakfast routine in mothers, stratified by gender.

Mothers’ Foods and Beverage Groups Consumption at Breakfast (Times per Week)	Boys’ Mother Breakfast Routine			Girls’ Mother Breakfast Routine		
	Occasional Breakfast Consumers	Breakfast Consumers		Occasional Breakfast Consumers	Breakfast Consumers	
	Mean (95% CI)	Mean (95% CI)	*p* *	Mean (95% CI)	Mean (95% CI)	*p* *
Fruits, berries and vegetables	2.35 (2.19, 2.51)	2.71 (2.63, 2.80)	**<0.001**	2.25 (2.10, 2.40)	2.77 (2.69, 2.84)	**<0.001**
Fruit juices fresh unsweetened	1.49 (1.36, 1.61)	1.63 (1.57, 1.69)	**0.048**	1.39 (1.27, 1.51)	1.63 (1.57, 1.69)	**<0.001**
Soft drinks sugar juices	1.08 (0.99, 1.17)	1.10 (1.02, 1.01)	**0.043**	1.10 (1.02, 1.18)	0.93 (0.89, 0.98)	**<0.001**
Milk or dairy products unsweetened	3.16 (2.99, 3.33)	4.15 (4.07, 4.24)	**<0.001**	3.14 (2.98, 3.31)	4.01 (3.93, 4.09)	**<0.001**
Milk or dairy products sweetened	1.55 (1.40, 1.70)	1.98 (1.91, 2.06)	**<0.001**	1.60 (1.46, 1.74)	1.94 (1.87, 2.01)	**<0.001**
Sweet or salty pastries	1.87 (1.75, 1.98)	1.92 (1.86, 1.97)	0.450	1.83 (1.73, 1.94)	1.84 (1.78, 1.89)	0.987
Low fiber cereal products	2.11 (1.96, 2.26)	2.44 (2.37, 2.52)	**<0.001**	2.08 (1.94, 2.22)	2.38 (2.31, 2.45)	**<0.001**
Whole-grain cereal products	2.15 (1.99, 2.31)	3.44 (3.36, 3.52)	**<0.001**	2.14 (1.99, 2.29)	3.44 (3.37, 3.52)	**<0.001**

* Statistical significance among different breakfast patterns. Significant *p* values are shown in bold font. Abbreviations: 95% CI, 95% confidence intervals. All analyses were adjusted by the following covariates: country, maternal education and mother BMI. Breakfast consumers categories: Occasional breakfast consumers: mothers who usually consume breakfast 2–5 days/week; Breakfast consumers, mothers who usually consume breakfast 6 or 7 days/week.

**Table 7:** Frequency of foods and beverages consumption at breakfast between mothers and their children.

		Boys’ Food and Beverage Consumption				Girls’ Food and Beverage Consumption			
		Never	Sometimes	Always		Never	Sometimes	Always	
		*n* (%)	*n* (%)	*n* (%)	*p* *	*n* (%)	*n* (%)	*n* (%)	*p* *
Fruits, berries, and vegetables									
Mothers’ food and beverage consumption	Never	1132 (71.3)	252 (16.7)	89 (9.3)	**<0.001**	1021 (72,1)	274 (16.5)	91 (8.6)	**<0.001**
	Sometimes	312 (19.7)	966 (63.8)	298 (31.3)		295 (20.8)	1091 (65.8)	347 (32.8)	
	Always	143 (9.0)	295 (19.5)	566 (59.4)		100 (7.1)	292 (17.6)	620 (58.6)	
Fruit juices fresh unsweetened									
Mothers’ food and beverage consumption	Never	1780 (85.4)	392 (26.8)	82 (16.8)	**<0.001**	1810 (85.1)	392 (25.8)	66 (14.8)	**<0.001**
	Sometimes	252 (12.1)	961 (65.8)	173 (35.5)		257 (12.1)	1032 (68.0)	146 (32.7)	
	Always	53 (2.5)	107 (7.3)	232 (47.6)		60 (2.8)	94 (6.2)	235 (52.6)	
Soft drinks and sugar juices									
Mothers’ food and beverage consumption	Never	2726 (93.9)	383 (44.8)	65 (29.3)	**<0.001**	2799 (93.5)	371 (45.1)	58 (29.4)	**<0.001**
	Sometimes	150 (5.2)	422 (49.4)	60 (27.0)		168 (5.6)	412 (50.1)	47 (23.9)	
	Always	27 (0.9)	50 (5.8)	97 (43.7)		26 (0.9)	40 (4.9)	92 (46.7)	
Milk or dairy products unsweetened									
Mothers’ food and beverage consumption	Never	355 (44.2)	172 (12.2)	173 (9.5)	**<0.001**	361 (46.6)	208 (13.2)	130 (7.3)	**<0.001**
	Sometimes	219 (27.2)	869 (61.9)	434 (23.8)		225 (29.0)	975 (61.9)	451 (25.5)	
	Always	230 (28.6)	364 (25.9)	1216 (66.7)		189 (24.4)	393 (24.9)	1188 (67.2)	
Milk or dairy products sweetened									
Mothers’ food and beverage consumption	Never	1241 (88.1)	634 (42.4)	340 (32.1)	**<0.001**	1288 (88.2)	664 (41.2)	294 (30.4)	**<0.001**
	Sometimes	127 (9.0)	756 (50.6)	240 (22.6)		131 (9.0)	829 (51.5)	223 (23.1)	
	Always	40 (2.8)	104 (7.0)	480 (45.3)		41 (2.8)	117 (7.3)	449 (46.5)	
Sweets or salty pastries									
Mothers’ food and beverage consumption	Never	969 (81.9)	467 (21.3)	105 (16.1)	**<0.001**	1037 (83.2)	490 (21.2)	87 (15.5)	**<0.001**
	Sometimes	188 (15.9)	1623 (74.1)	266 (40.7)		191 (15.3)	1701 (73.6)	237 (42.2)	
	Always	26 (2.2)	99 (4.5)	283 (43.3)		18 (1.4)	121 (5.2)	237 (42.2)	
Low fiber cereal products									
Mothers’ food and beverage consumption	Never	685 (76.7)	637 (31.7)	240 (21.2)	**<0.001**	680 (75.3)	616 (28.3)	188 (18.8)	**<0.001**
	Sometimes	161 (18.0)	1187 (59.1)	306 (27.1)		187 (20.7)	1357 (62.3)	322 (32.2)	
	Always	47 (5.3)	183 (9.1)	585 (51.7)		36 (4.0)	204 (9.4)	489 (48.9)	
Whole-grain cereal products									
Mothers’ food and beverage consumption	Never	954 (63.5)	176 (11.6)	63 (6.2)	**<0.001**	873 (60.5)	184 (10.9)	57 (5.9)	**<0.001**
	Sometimes	306 (20.4)	935 (61.5)	227 (22.3)		354 (24.5)	1088 (64.7)	163 (16.9)	
	Always	242 (16.1)	410 (27.0)	727 (71.5)		217 (15.0)	409 (24.3)	743 (77.2)	

* Significant *p* values are shown in bold font. Frequency of food and beverage groups’ consumption: Never: 0 times/week; Sometimes: 1–4 times/week; Always: 5–7 times/week.

## Data Availability

The datasets generated and/or analyzed during the current study are not publicly available, since the data used is confidential based on Feel4Diabetes publications rules, but are available from the corresponding author on reasonable request.
